# Endodontic and dental implant treatment: key considerations and comparisons

**DOI:** 10.1038/s41415-025-8337-8

**Published:** 2025-05-23

**Authors:** Bhavin Bhuva, Pareet Shah, Francesco Mannocci

**Affiliations:** 026667951306239451598 Honorary Clinical Lecturer, King´s College London, UK; Consultant in Endodontics, Guy´s Hospital, Guy´s and St Thomas´ NHS Trust, UK; Specialist in Endodontics, Private Practice, United Kingdom; 078089782677701312189 Senior Clinical Lecturer and Deputy Director Implant MSc, UCL Eastman, London, UK; Specialist in Prosthodontics, Private Practice, United Kingdom; 413652267787348569581 Professor and Head of Endodontology, King´s College London, UK; Specialist in Endodontics and Restorative Dentistry, Private Practice, United Kingdom

## Abstract

There remains huge variability in decision-making when it comes to whether a compromised tooth requiring endodontic treatment should be saved, or extracted and replaced with an implant. Both internal and external biases, as well as inconsistent data from clinical studies, further complicate this frequent clinical conundrum. This paper presents tangible outcomes for both root-filled teeth and dental implants, together with comparative research to help clinicians better understand the available data. It is hoped that we can also highlight the key considerations when treatment planning for both root-filled teeth and dental implants. Both treatment modalities have excellent survival rates, but at the same time, neither is a panacea. Holistic and thoughtful consideration is required to help guide patients to make well-informed choices regarding their treatment.

## Key points

- Both root-filled teeth and dental implants have excellent success and survival rates. Clinicians should appreciate the difference between success and survival when evaluating the available literature.
- A holistic treatment planning approach is required when decision-making between retaining a compromised root-filled tooth and replacing it with a dental implant.
- Clinicians should be aware of short-, medium- and long-term complications for both root-filled teeth and dental implants. The role of occlusion appears critical to the survival of both treatment modalities.

## Introduction

Dentists and patients are often faced with the complex decision of when to save, or when to extract, a compromised tooth. Making the choice between trying to retain a structurally compromised tooth by way of endodontic (and/or periodontal) and restorative treatment versus extracting the tooth and replacing it requires thorough examination, thoughtful treatment planning and discussion.

There are many factors, both clinical and patient-centred, to consider when evaluating a compromised tooth. The remaining tooth structure, periodontal support and endodontic status must be assessed. In addition, factors such as the strategic importance of the tooth, its location, occlusal factors, aesthetics and cost must also be considered. Furthermore, the patient's perspective on the treatment processes, as well as the case specifics of alternative options for tooth replacement, will differ for each patient. Taking into account the multitude of considerations, and moreover, communicating this information in a clear way, also brings its own challenges.

To assist clinicians in evaluating the compromised tooth, several systematic assessment indices have been developed. Tools such as the Restorability Index^1^ facilitate evaluation of the residual tooth structure, whilst The British Endodontic Society Case Assessment Tool^2^ can be used to determine the case complexity before endodontic treatment. More holistic evaluation of a tooth can be carried out using indices such as The Dental Practicability Index, which remains the only tool which has been validated by a clinical study, albeit limited to the evaluation of teeth undergoing root canal retreatment (Table 1).^3^ Both indices and tools have been shown to provide helpful guidance to assess tooth prognosis in an objective and tangible way. While the use of tools and indices facilitate a systematic and logical analysis of each case, restorative indices in particular are susceptible to the limitations of accurately quantifying tooth structure loss. Therefore, these indices should never be used in isolation, or to override clinical judgement and experience.

**Table 1:** The Dental Practicality Index can be used to assess the prognosis of a tooth in four domains: endodontic, periodontal, prosthodontic, and importantly, in context of the patient's medical and dental conditions. Each domain is weighted according to complexity. The total gives an indication of when to treat, refer, or extract a compromised tooth. The index has been validated in clinical research, both with regards to decision making and treatment outcomes. Reproduced with permission from S. Patel *et al*.,' The Dental Practicality Index - to treat or not to treat', *British Dental Journal*, vol 236, 2024, Springer Nature^81^

**Weighting**	**Endodontic treatment need**	**Periodontal treatment need**	**(Structural) integrity**	**Context**
0: No treatment required	Healthy periapical status	Healthy periodontal status	Sound coronal status	Local: Isolated dental problems where adjacent teeth are healthy General: Replacing of a strategic tooth may be excessively complex History of intravenous bisphosphonates, head and neck radiotherapy
1: Simple treatment required	Accessible root canal system (eg radiographically easily identifiable root canal[s], easily retrievable root canal filling material)	Clinical attachment Loss with pocketing <4 mm and bone loss up to coronal third (eg part of a picture of poor periodontal maintenance where conventional professional mechanical plaque removal is required with or without the administration of local anaesthetic)	Straightforward direct or indirect restoration required	Local: Prosthodontic treatment planned of neighbouring teeth which may influence treatment plan for tooth being assessed Tooth to be used as a bridge abutment Terminal tooth General: Poor oral maintenance Radiotherapy of head/neck planned Immunocompromised patient
2: Complex treatment required (consider referral)	Challenging/complex root canal system (eg sclerosed root canal, acute curvatures, internal/external cervical resorption, incomplete fracture) Complex re-root canal treatment (eg fracture instrument removal, blocked canal[s], perforations) Difficulty in obtaining profound anaethesia	Clinical attachment loss with pocketing >4+ mm and bone loss to the mid-third of the root (eg professional mechanical plaque removal with the administration of local anaesthetic, or more complex surgery with or without a graft procedure)	Limited (<30%) sound, coronal tooth volume and/or inadequate ferrule. May require post- and/or crown lengthening	Local: Prosthodontic treatment planned of multiple teeth, including adjacent teeth General: High caries rate Parafunctional habits Dry mouth Stage III periodontitis: severe disease with potential for additional tooth loss^82^
6: Impractical to treat	Untreatable root canal system Impractical to re-treat (eg complete fracture, inability to achieve patency in a symptomatic tooth)	Untreatable or rapidly progressing periodontal condition (eg bone loss to the apical third with pocketing >4+ mm)	Insufficient coronal tooth volume to support a restoration and/or inadequate ferrule	Local: Retention of the tooth being assessed would constrain and/or compromise an otherwise simple and predicable treatment plan (for example, extensive dental or implant bridgework) General: (Potentially) life threatening medical conditions which should be managed in tertiary care. Stage IV advanced periodontitis with extensive tooth loss and potential for loss of dentition^82^

Over recent years, the number of dental implants being placed has increased markedly,^4^ with the treatment also being provided by a growing number of clinicians. Both soft and hard tissue augmentation techniques have evolved and are now commonplace, facilitating implant placement where this may have been considered unfeasible in the past. However, with the greater provision of dental implants, there are also now more data on their mechanical and biological complications.

When faced with the decision of whether to save a compromised tooth, or extracting it and considering implant replacement, a good understanding of the treatment outcomes for both modalities is required. To compare outcomes, we first need to understand that there are differences in how these are evaluated, and that even within each modality, varying parameters are used.

Success for root canal treatment has historically been based on the resolution or prevention of apical periodontitis. This is associated with the absence of clinical signs or symptoms and radiographic healing of periapical disease using conventional radiographic imaging, and/or more recently, cone beam computed tomography (CBCT). Guidelines suggest that clinical and radiographic follow-up should be carried out one year after the completion of treatment, and thereafter, on a yearly basis for four years.^5^ More recently, and discussed shortly, research has shifted towards assessing tooth survival and/or function (Table 2).

**Table 2:** Terms and definitions of criteria used to describe treatment outcomes for root-filled teeth

**Outcome measure**	**Definition**	**Author**
Success	Absence of clinical signs and symptoms No evidence of periapical disease (intact periodontal ligament space and lamina dura).	Strindberg L Z. The dependence of the results of pulp therapy on certain factors: an analytic study based on radiographic and clinical follow-up examinations. *N G Mauritzons Boktryckerl* 1956.
Survival	Tooth present and potentially functional at time of recall, regardless of the clinical or radiographic findings (Ng *et al.* 2008)^9^ Tooth in question has absence of any signs or symptoms independent of the PAI score.	Ng Y-L. *Factors affecting outcome of non-surgical root canal treatment.* London: University College London, 2008. Friedman S, Mor C. The success of endodontic therapy - healing and functionality. *J Calif Dent Assoc* 2004; **32:** 493-503.
Function	A treated tooth or root that is serving its intended purpose in the dentition.	The American Association of Endodontists Communique. *AAE and Foundation approve definition of endodontic outcomes*. 2005; **XXIX:** 3.
Patient-centred outcomes	17 questions, aiming to capture seven conceptually formulated dimensions of oral health adapted from for endodontic treatment: Functional limitation, physical pain, psychological discomfort, physical disability, psychological disability, social disability, and handicaps (Slade & Spencer 1994).^83^	Dugas N N, Lawrence H P, Teplitsky P, Friedman S. Quality of life and satisfaction outcomes of endodontic treatment. *J Endod* 2002; **28:** 819-827.

For dental implant treatment, success has been defined as ‘the element (implant or reconstruction) being present at the follow-up examination, and complications are absent', while survival has been specified as ‘the element (implant or reconstruction) is present at the follow-up examination, but its condition is not specified'. These criteria are both frequently cited when discussing implant outcomes. However, numerous iterations of these terms have been used to include, or exclude, clinical presentations (for example, the health of the peri-implant soft tissues) and acceptable levels of bone loss (Table 3).

**Table 3:** Terms and definitions of criteria used to describe treatment outcomes for dental implants

**Outcome measure**	**Definition**	**Author**
Success	No mobility No nerve lesion No signs of inflammation (probing depths over 4 mm are considered comparable to infection) Vertical bone loss is less than 0.2 mm/year after the first year of placement No peri-implant radiographic translucency.	Albrektsson T, Zarb G, Worthington P, Eriksson A R. The long-term efficacy of currently used dental implants: a review and proposed criteria of success. *Int J Oral Maxillofac Implants* 1986; **1:** 11-25.
Success Survival	Success: the element (implant or reconstruction) is present at the follow-up examination, and complications are absent (this is defined as chair time being required after incorporation of the prosthesis). Survival: the element (implant or reconstruction) is present at the follow-up examination but its condition is not specified.	https://www.iti.org/academy/consensus-database/consensus-statement/-/consensus/implant-survival-and-complications/1321
Success	No pain or tenderness upon function No mobility <2 mm radiographic bone loss after complete bone remodelling and a probing depth ≤4 mm (no bleeding and/or suppuration on probing) No implant thread exposure Acceptable occlusion.	Misch C E, Perel M L, Wang H L *et al*. Implant success, survival, and failure: the International Congress of Oral Implantologists (ICOI) Pisa Consensus Conference. *Implant Dent* 2008; **17:** 5-15.
Success	No clinical signs of inflammation (eg gingival erythema, oedema, and changes in soft tissue consistency) Absence of bleeding and/or suppuration on gentle probing No increased probing depths No radiographic evidence of progressive bone loss. A secondary case definition was proposed, in the absence of longitudinal data, that includes bleeding on probing and/or suppuration on probing at ≥1 site, probing depth ≥6 mm, and bone loss ≥3 mm).	Papapanou P N, Sanz M, Buduneli N *et al*. Periodontitis: Consensus Report of Workgroup 2 of the 2017 World Workshop on the Classification of Periodontal and Peri-Implant Diseases and Conditions. *J Periodontol* 2018; **89:** 173-182.
Patient-centred outcome	49-item questionnaire (modifications, 14 or 17 questions), aiming to capture seven conceptually formulated dimensions of oral health: functional limitation, physical pain, psychological discomfort, physical disability, psychological disability, social disability, and handicaps (Slade & Spencer 1994).^83^	Allen P F, McMillan A S, Walshaw D, Locker D. A comparison of the validity of generic- and disease-specific measures in the assessment of oral health-related quality of life. *Community Dent Oral Epidemiol* 1999; **27:** 344-352.

To provide more meaningful and tangible criteria for implant outcomes, a health scale has been developed, similar to those used for natural teeth, which provides more specific details on treatment outcome, ranging from success, satisfactory survival, compromised survival and failure (Table 4).^6^

**Table 4:** The International Congress of Oral Implantologists (ICOI) Health Scale for dental implants. Formulated to provide an index like those used for natural teeth but with specific criteria relevant to dental implants. Reproduced with permission from C. E. Misch *et al*., ‘Implant success, survival, and failure: the International Congress of Oral Implantologists (ICOI) Pisa Consensus Conference', *Implant Dentistry*, vol 17, 2008, Lippincott Journals^84^

Implant Quality Scale Group*	**Clinical conditions**
I. Success (optimum health)	a) No pain or tenderness upon function b) 0 mobility c) <2 mm radiographic bone loss from initial surgery d) No exudate history
II. Satisfactory survival	a) No pain on function b) 0 mobility c) 2-4 mm radiographic bone loss d) No exudate history
III. Compromised survival	a) May have sensitivity on function b) No mobility c) Radiographic bone loss >4 mm (less than 1/2 of implant body) d) May have exudate history
V. Failure (clinical or absolute failure)	a) Pain on function b) Mobility c) Radiographic bone loss >1/2 length of implant d) Uncontrolled exudate e) No longer in mouth
*International Congress of Oral Implantologists, Pisa, Italy, Consensus Conference, 2007	

As survival criteria for both root-filled teeth and dental implants are fairly similar, when compared to other outcome metrics, comparative studies assessing survival allow clinicians to differentiate the outcomes for both more objectively. These findings will be discussed in a later section on comparative research.

This review aims to discuss the key decision-making considerations and outcomes for both endodontic and implant treatment.

## Outcomes for root-filled teeth

The findings of studies relating to endodontic outcome measures vary according to the criteria used. Success rates have been shown to be as high as 85.2−92.6%^7^^,^^8^ for primary root canal treatment and 77.2−83%^9^^,^^10^ for root canal retreatment.

However, despite these excellent ‘biological' outcomes, more recently, the focus has shifted towards the ‘survival' of root-filled teeth, as contextually, this is perhaps more meaningful for patients when making treatment planning decisions. Data from studies relating to the survival, rather than the success, of root-filled teeth provides valuable information on causes of failure, as well as allowing direct comparison with other treatment modalities, such as implant treatment.^11^ This also helps patients make informed choices on the best treatment for themselves.^12^

The available studies suggest that the causes for the loss of root-filled teeth are often biomechanical, rather than biological, in aetiology. Therefore, aside from post-treatment endodontic disease, root-filled teeth may also fail due to unrestorable caries, restorative failure, irretrievable cusp or crown fracture, vertical root fracture, or periodontal disease.^13^

## Considerations for root-filled teeth

### Patient factors

The impact of systemic health, diseases and medicine on the outcome of root canal treatment appears to be an emerging area of interest.^14^ It has been postulated that specific health conditions and/or medications may affect healing by inhibiting fibroblast function and/or bone turnover. Furthermore, the vascularity to the periapical tissues may be impaired, reducing the provision of nutrients to the area.

Much of the research has studied the effect of diabetes mellitus on endodontic healing outcomes. While some researchers have failed to find a correlation,^15^ others have demonstrated inferior outcomes.^14^^,^^16^ In addition to finding a relationship between diabetes mellitus and inferior endodontic outcomes, steroid therapy has also been associated with lower success rates.^17^ However, overall, the observations for root-filled teeth are weaker than those for dental implants, as failures are more frequently related to biomechanical causes.

Anti-resorptive drugs (ARDs) do not appear to affect the outcome of root canal treatment. In fact, given the associated risks of implant placement in patients on these medications, the decision to save a compromised tooth may be heavily influenced by this consideration.^18^

### Clinical factors

#### Periapical status

**Fig. 1:**
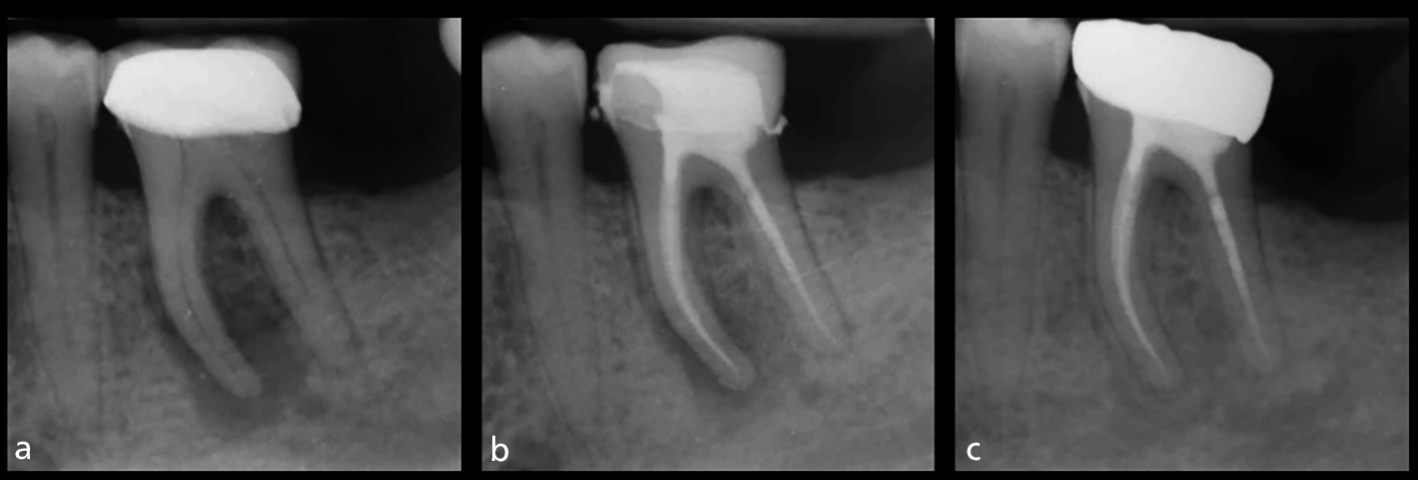
a, b) Primary root canal treatment of this mandibular left first molar was carried out following crown removal and restorability assessment. A new crown was placed following the completion of root canal treatment. c) At the one-year follow-up, there has been complete clinical and radiographic healing, indicating a successful outcome

Most of the literature indicates that the presence of a pre-operative periapical radiolucency is the most important clinical factor affecting the success rate of root canal treatment, both dependently^19^, and independently, of pulpal status.^20^ Overall, the success rates for teeth without periapical disease are approximately 9-13% higher than for those with radiographic disease (Fig. 1).^7^

For teeth requiring root canal retreatment, the presence of a pre-operative periapical radiolucency appears to have an even greater influence on treatment outcome.^21^ In a systematic review, evaluating outcome studies which included the pre-operative status, the weighted success rates for teeth with pre-operative radiolucencies were 28% lower than those with an absence of periapical disease.^22^

#### Residual tooth structure

Several authors have suggested that the amount of residual tooth structure left after endodontic-restorative procedures is critical to their survival. Researchers have evaluated the amount of remaining tooth structure in several ways, which include, the presence of a ferrule effect,^23^ the number of remaining walls^24^ and more recently, residual tooth volume.^25^

**Fig. 2:**
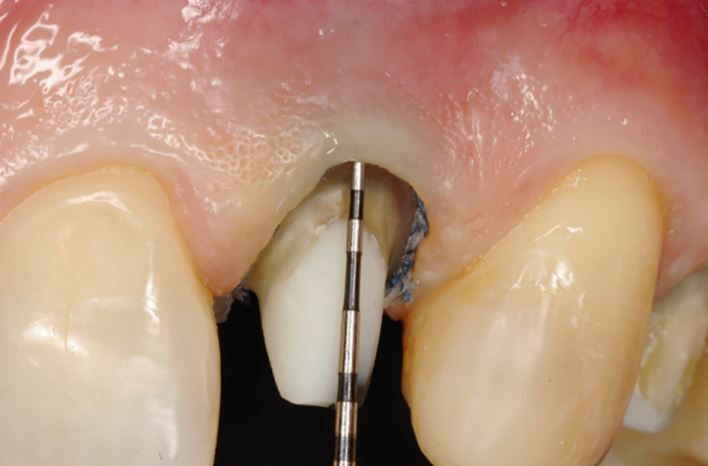
Even in compromised teeth, an adequate ferrule effect can often be obtained. In this case, there is more than 2 mm of circumferential dentine coronal to the crown margin which will ensure that both occlusal and non-occlusal forces are not undesirably transferred to the fibre post and composite core complex which has been placed. Root-filled teeth with an adequate ferrule effect have been clearly shown to perform more favourably than those that do not have sufficient circumferential dentine height. When an adequate ferrule effect cannot be obtained, the prognosis should be considered; crown lengthening or orthodontic extrusion may be options if the patient is motivated to retain their natural tooth. Reproduced with permission from F. Mannocci *et al*., *Adhesive restoration of endodontically treated teeth*, 2008, Quintessence Publishing^80^

The ferrule effect refers to the circumferential collar of cast metal or ceramic (provided by the cuspal coverage restoration), which encircles near parallel walls of dentine, coronal to the margins of the cuspal coverage preparation (Fig. 2). A minimum height of 2 mm of circumferential supra-marginal dentine has been suggested. The presence of a ferrule effect is widely considered to be a key determinant for the long-term survival of all tooth types. A number of laboratory studies have demonstrated superior biomechanical performance of root-filled teeth when there is an adequate ferrule effect.^26^ The failure rate for 87 root-filled teeth (including all tooth types) were assessed in a prospective clinical trial with a three-year observation period.^27^ Classification of the teeth was made according to whether they had a ferrule height of greater or less than 2 mm. The authors found that the overall restoration failure rate was 16.1%. However, failure rates of 26.2% for teeth with less than 2 mm of ferrule height, and 6.67% for teeth with greater than 2 mm were observed.

The survival of root-filled molar teeth with respect to the number of residual walls with a minimum thickness of 2 mm has also been studied.^28^ After five years, 78% of teeth in the group with ‘maximum residual tooth structure', 45% of teeth with ‘moderate tooth structure' and only 18% of teeth with ‘minimal tooth structure' survived. It is important to acknowledge that a limitation of this study was that none of the included teeth were restored with cuspal coverage restorations. However, similar outcomes have been reported in studies when this has been carried out.^29^

#### Tooth location and proximal contacts

**Fig. 3:**
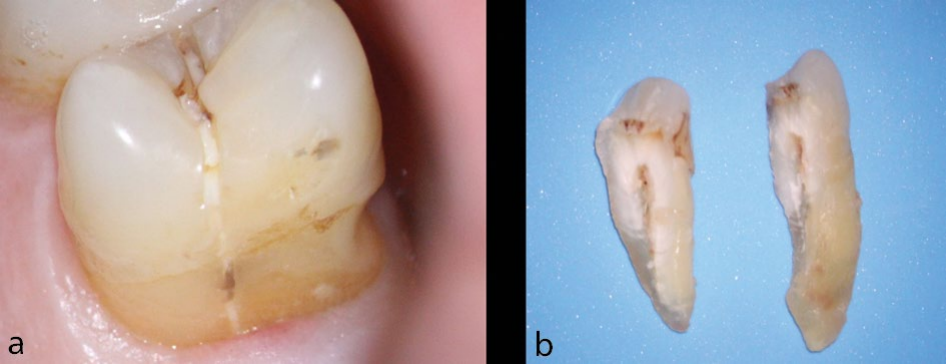
The combination of missing proximal contacts and parafunction cannot be understated. a, b) A completely unrestored maxillary left second premolar has ‘split' following separation of a vertical root fracture. In patients demonstrating such fractures, the prognosis of any treatment at other sites, including root canal and implant treatment, should be carefully considered

Compromised root-filled teeth are at greater risk of structural failure because of tooth structure loss, and the biomechanical consequences of endodontic and restorative treatment. In addition, the effect of functional and/or parafunctional stresses imparted on root-filled teeth are, in part, related to both tooth location and the presence of proximal contacts (Fig. 3).

**Fig. 4:**
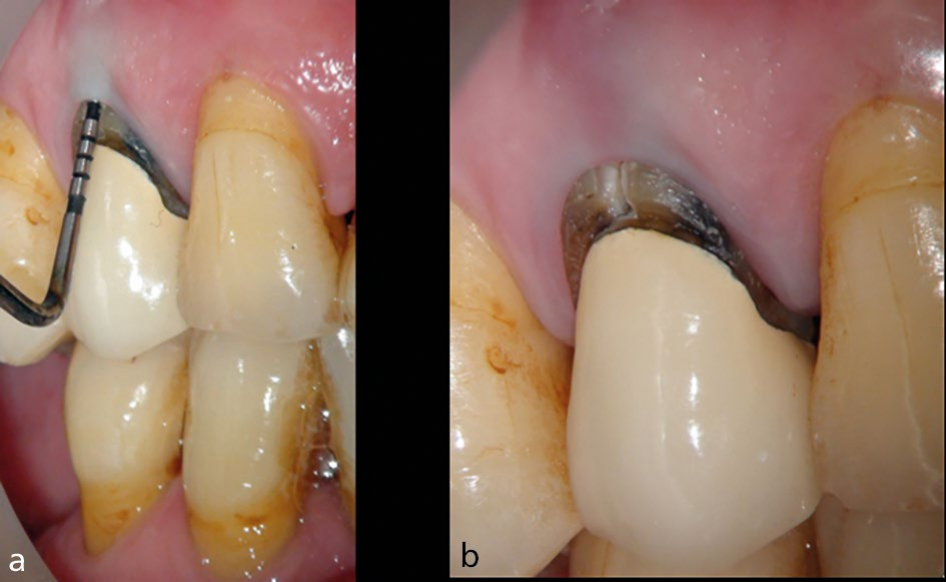
A root-filled maxillary right first premolar has developed buccal tenderness several years after endodontic treatment and crown placement. a) A deep buccal probing depth is evident. b) A vertical root fracture is observed. Root-filled teeth are susceptible to biomechanical complications, and in this case, it is important to acknowledge that this is the last functioning tooth in the arch, indicating that it has been subjected to increased occlusal and non-occlusal loading

**Fig. 5:**
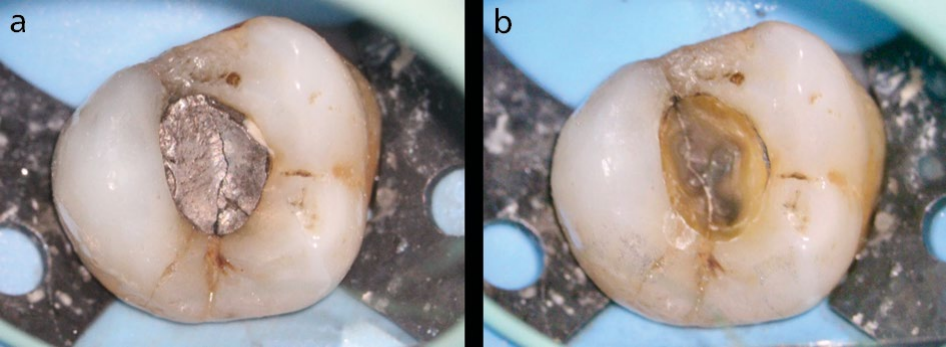
This maxillary left second molar has no proximal contacts. a, b) Despite being fairly minimally restored and having four residual walls, a mesial-to-distal crack line has developed. When considering the prognosis of this tooth, the absence of neighbouring teeth must be considered alongside the extent of the crack. If the tooth is to be retained, expedient cuspal coverage and careful occlusal assessment/management are necessary

A retrospective study with an observation period of between 4-10 years,^17^ and a prospective study with a 2-4-year observation period^30^ demonstrated that the last functioning teeth in the arch (Fig. 4), and those without neighbouring contacts (Fig. 5), had inferior survival to non-terminal teeth^31^ and those with neighbouring contacts, respectively.^32^ Root-filled second molar teeth have been shown to have the greatest likelihood of catastrophic failure requiring extraction.^33^

#### Restoration type/cuspal coverage

Irretrievable crown or root fracture is a significant risk to the survival of compromised root-filled teeth. Furthermore, an optimal coronal seal reduces the risk of microleakage which may lead to reinfection of the root canal system. Cuspal protection of root-filled posterior teeth (premolars and molars) has been shown to improve survival, reducing the risk of biomechanical complications.^33^

**Fig. 6:**
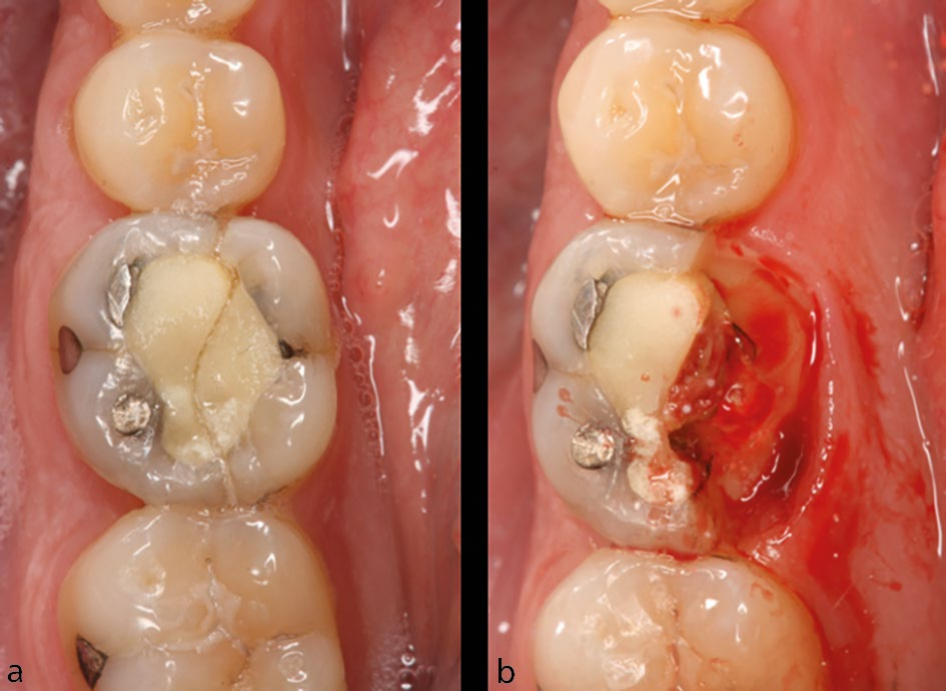
This root-filled mandibular left first molar tooth was not expediently restored with a cuspal coverage restoration following root canal treatment. a) Subsequently, there has been a significant oblique fracture of the lingual cusps. b) Following removal of the mobile portion of tooth, the fracture margin was found to be extensively subgingival, resulting in a poor prognosis. The decision was made to extract the tooth

The expediency of definitive cuspal coverage restoration placement is also an important factor affecting tooth survival (Fig. 6). An eight-year retrospective study of root-filled posterior teeth showed that those restored within four months of the completion of root canal treatment were three times less likely to be extracted than those restored after this time.^32^

In a large retrospective study, 1,273 root-filled teeth were followed for up to 25 years.^34^ The authors concluded that long-term survival was significantly improved when cuspal coverage restorations were placed. A further retrospective database study, carried out in a specialist endodontic clinic, assessed the survival of all tooth types following root canal treatment and concluded that teeth restored with crowns had a higher estimated survival rate (91%) than those restored with direct restorations (76%).^35^ With respect to posterior root-filled teeth specifically, an eight-year retrospective study found that teeth restored with crowns had a survival rate of 71%, compared with a survival rate of only 58% for teeth which did not receive cuspal coverage restorations.^32^ Aquilino and Caplan observed that root-filled teeth that did not have crowns were extracted six times more frequently than those with crowns.^33^

#### Occlusal factors

There is little evidence in relation to the effects of parafunctional activity on the survival of root-filled teeth. However, the structural and biomechanical effects of endodontic-restorative treatment predispose them to the consequences of both excessive functional and non-functional forces.^36^ Furthermore, parafunction and/or non-working side interferences can lead to undesirable non-axial loading, which will in turn, increase the risk of vertical root fracture.^37^^,^^38^ It is evident that vertical root fractures are far more common in root-filled posterior teeth.^39^^,^^40^ It has also been shown that the further a tooth is to the end of the arch, the higher functional and non-functional occlusal loading is likely to be.^41^^,^^42^ To compound these predisposing factors further, loading on root-filled teeth may be affected by the loss of pulpal proprioception.^43^ It is clear that the occlusion of teeth requiring endodontic-restorative treatment (particularly those which are structurally compromised/or those presenting with cracks) should be carefully assessed and managed to prevent excessive non-axial and/or parafunctional forces.

#### Cracked teeth

The diagnosis and prognostication of cracked teeth remains a challenge for most clinicians. Cracks are common findings, particularly in mandibular (terminal) molar teeth. A practice-based network study found that only 1% of teeth with cracks required root canal treatment in the three years following detection.^44^ However, cracks in teeth requiring root canal treatment should be considered as a significant factor affecting their survival. Based on emerging research on the survival of root-filled teeth with cracks, the location and extent of cracks should not be considered in isolation when determining the prognosis. Given the biomechanical effects of endodontic and restorative treatment, it is likely that teeth undergoing root canal retreatment are even more likely to present with, or develop, cracks or fractures.

A systematic review assessing the survival of cracked root-filled teeth reported a 48-month survival rate of 89.6% and a 60-month survival rate of 84%.^45^ Cumulative analysis demonstrated that cracks were most prevalent in mandibular molars. The key prognostic factors affecting survival were multiple cracks, radicular cracks and probing depths greater than 3 mm associated with the crack. However, there was only an 8-9% increase in incidence of extraction for teeth exhibiting all three of these risk factors.

A prospective cohort study assessed both the success and survival rate of root-filled premolar and molar teeth exhibiting cracks with radicular extension over 2-4 years.^46^ In this study, the included teeth had cracks extending from the canal orifice to at least 5 mm into the canal. Furthermore, all included teeth had increased periodontal probing depths associated with the crack. Cracks of this nature and depth would be considered to have poor to hopeless prognoses by most clinicians. The protocol for managing the cracks in this study used a novel fluoride-releasing resin with pre-reacted nano-glass particles, which was placed in the canal, coronal to the root filling, and below the level of the visible crack. Following this, the teeth were restored with cuspal coverage restorations. Of the 70 teeth included in the study, 53 and 59 teeth, respectively, were available for success and survival follow-up. After an observation period of two years, 100% of the teeth survived, while at four years, 96.6% were still present. The authors reported that the depth of the cracks did not appear to impact prognosis; however, the depth of the periodontal probing depth associated with the crack was a significant prognostic factor. Interestingly, tooth location did not impact prognosis, with terminal teeth having similar outcomes to other teeth.

**Fig. 7:**
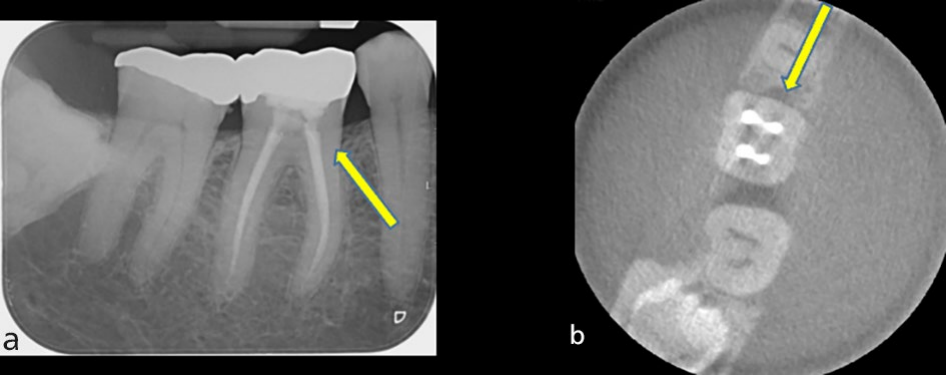
Five years after the completion of root canal treatment and crown placement, this mandibular right first molar has developed symptoms. No isolated periodontal probing depths were evident. a) Although, there is no evidence of emerging disease evident with conventional radiographic imaging. b) Small volume CBCT demonstrates a localised area of mesial crestal bone loss, consistent with a vertical root fracture. Image courtesy of Dr Noushad Rahim

**Fig. 8:**
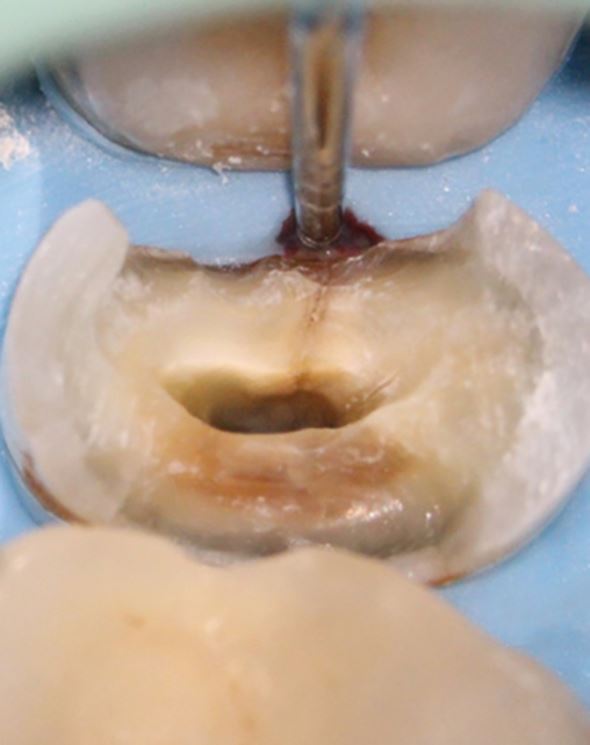
Following coronal disassembly, a mesial crack line was observed in this maxillary right second premolar. At this stage, it is possible to carry out comprehensive periodontal probing of the affected surface. A narrow-isolated probing depth, such as that observed in this case, is consistent with a vertical root fracture. As these often occur in the interproximal region, it is not always possible to detect narrow pockets such as these until the coronal restoration has been removed

An important consideration for cracked root-filled teeth is that, in a number of cases, their eventual failure will be as a result of the crack progressing to a vertical root fracture.^47^ Early diagnosis of vertical root fractures can be very challenging due to the lack of symptoms and tangible clinical and radiographic changes (Fig. 7). Furthermore, as most corono-apical cracks develop mesially and distally (in the interproximal region), even when attachment loss has occurred, it can be difficult to determine this with periodontal probing (Fig. 8). In any event, careful monitoring is required to ensure that excessive bone loss does not occur when a vertical root fracture develops, as to ensure future implant placement is not compromised.^48^

It has been shown that vertical root fractures are more likely to develop in posterior teeth,^39^^,^^40^ while terminal root-filled teeth, and those without proximal contacts, are subject to higher loading during normal function and parafunction.^41^^,^^42^ In a four-year prospective study assessing the factors affecting tooth survival following root canal treatment, 68% of terminal teeth that were extracted exhibited fractures.^17^ Of these, 58% of teeth without proximal contacts were extracted due to fracture when compared with 38% of teeth with proximal contacts.

#### Root canal retreatment

**Fig. 9:**
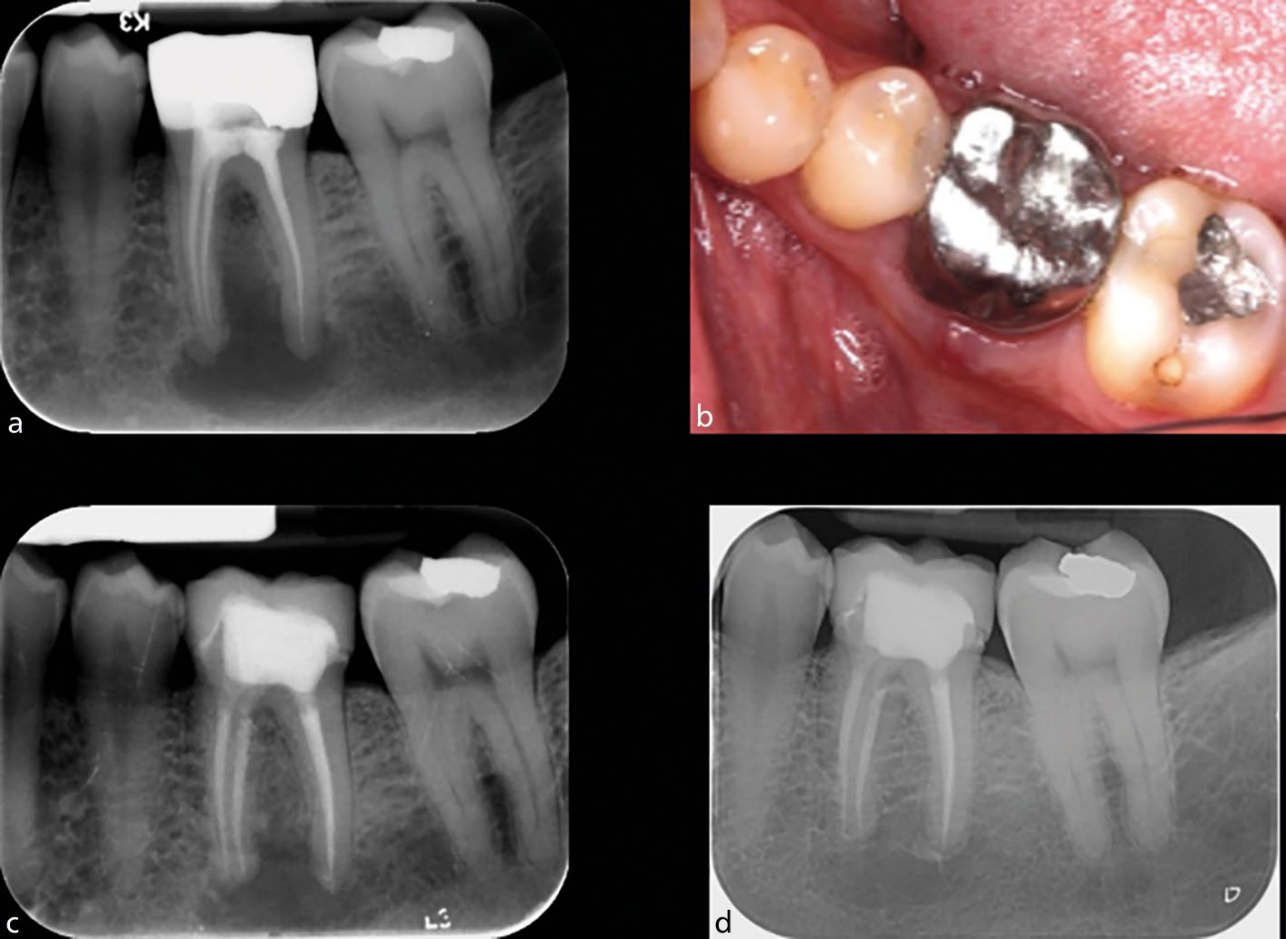
a, b, c) Root canal retreatment of this mandibular left first molar presenting with a large periapical lesion was carried out, after which a new crown was placed. d) At the ten-year review, clinical and radiographic examination reveals excellent healing. Image courtesy of Dr Kreena Patel

Studies assessing the success rates for root canal retreatment suggest inferior outcomes when compared to primary treatment. Systematic reviews report success rates of approximately 77-83% for secondary treatment (Fig. 9).^9^^,^^10^ However, the outcomes for retreatment appear to show much greater variability, and this appears to relate to the additional technical challenges that present in previously treated cases. The effect of these complicating factors is highlighted in a prospective study where the operators classified the teeth based on the preoperative radiograph into either ‘root canal anatomy respected' or ‘root canal anatomy altered' groups.^49^ The key differences between these two groups were that there were discernible complicating factors with respect to retreatment, such as transportation, ledge formation, calcification, instrument separation or perforation in the ‘root canal anatomy altered' group, while these were not evident in the ‘root canal anatomy respected' group.

A total of 452 teeth underwent retreatment in a single visit and healing was assessed two years postoperatively. The overall success rate was 69%. However, the success rate in the ‘root canal anatomy respected' group was 86.8% while it was only 47% in the ‘root canal anatomy altered' group.

With respect to the differences in survival of teeth undergoing primary or secondary root canal treatment, there are conflicting results. Ng *et al*. reported tooth survival following primary root canal treatment to be 95.4% and 95.3% for retreated teeth when performed by postgraduate students in a prospective study with 2-4-year follow-up.^17^ However, only 50% of the original cohort of patients were reviewed at the last recall, and the study had not been powered to measure the difference in survival between teeth which had undergone primary or secondary root canal treatment. In a large Taiwanese registry study, including over three million teeth, the survival rate for teeth undergoing primary treatment was higher (90.9%) than that for retreated teeth (88.4%) after five years.^50^

**Fig. 10:**
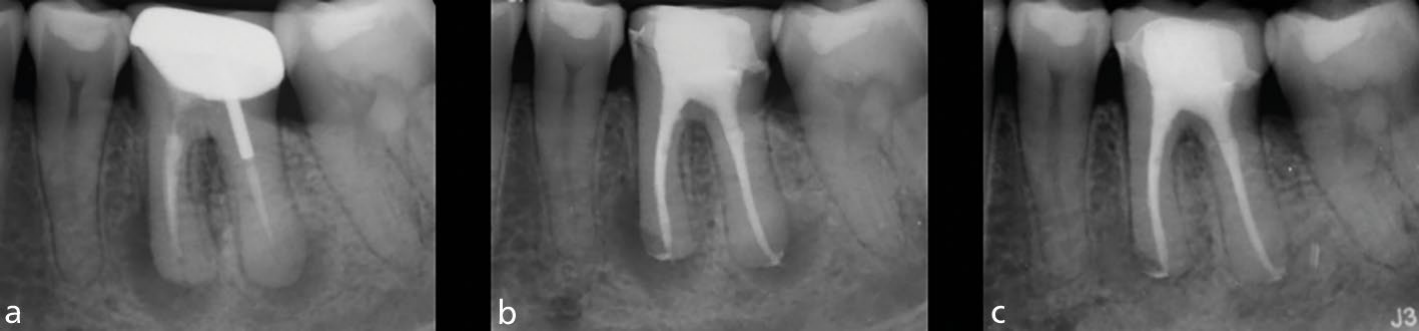
a) This mandibular left first molar had both restorative and endodontic considerations which were discussed in relation to the available treatment options. b) The patient elected to proceed with root canal retreatment, following restorability assessment. The crown and post were removed after which root canal retreatment was carried out. A composite core and adhesively cemented ceramic crown were placed. c) At the one-year review, good healing has been observed

The effect of residual tooth structure is, perhaps, even more relevant in compromised teeth undergoing root canal retreatment. It is often necessary to carry out coronal disassembly on teeth already restored with crowns, extensive cores and/or posts (Fig. 10). Therefore, the effect on the residual tooth structure may be even more impactful during these procedures. The survival of posterior teeth following root canal retreatment in relation to the percentage of remaining sound tooth volume (determined with three-dimensional digital scanning) has been prospectively studied.^25^ After one year, 137 posterior (premolar and molar) teeth were reviewed. Unfavourable complications were observed in 30.3% of teeth with less than 30% of residual tooth structure. When more than 30% of residual tooth structure was present, only 14.4% of teeth experienced complications. In a follow-up study, the same teeth were reassessed after four years.^3^ The percentage of teeth extracted when there was less than 29.5% of residual tooth structure was three times higher (12.5%) when compared with teeth where there was more than 29.5% of structure (3.5%).

## Outcomes for dental implant treatment

Endosseous, screw-type dental implants have been used for several decades for the management of partially dentate and edentulous patients. They are usually made of titanium or one of its alloys, with zirconia also being a valid alternative material for their fabrication.^51^

Dental implants are essentially a support mechanism for a prosthesis and should be considered alongside tooth-supported and mucosa-borne replacements for missing teeth. Increasingly, however, dental implants are often seen as the ‘method of choice' if factors such as bone volume, prosthetic space and patient health are favourable. As such, it is important for clinicians to discuss dental implants as a valid treatment option for patients, either when a tooth is already absent or when considering the options for a compromised tooth.

**Fig. 11:**
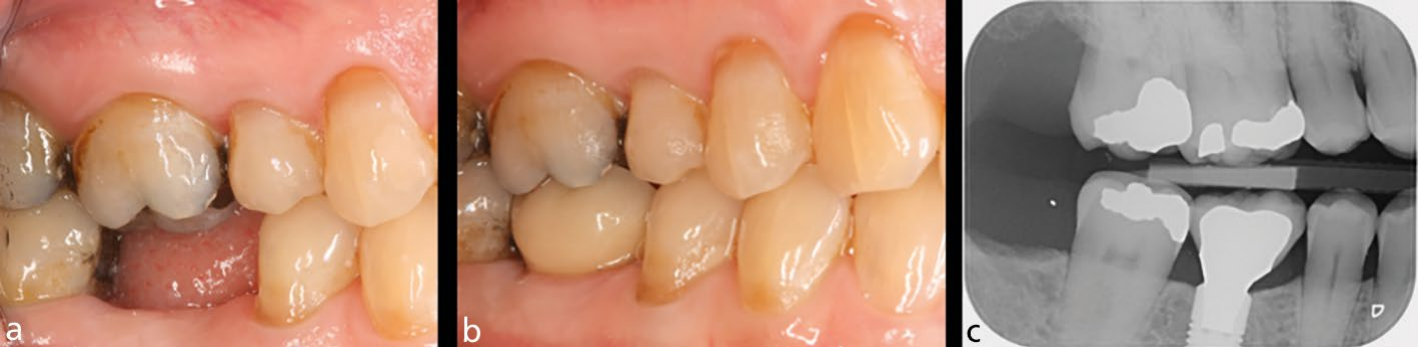
This patient presented with pain from a mandibular right first molar which had been root filled and restored with a crown ten years previously. It was decided to extract the tooth, due to an extensive perforation and bone loss in the furcation region. a) After the area had healed, the patient requested a fixed replacement. b) After discussion of the treatment options, a single tooth implant-supported crown was recommended. The implant was placed and restored with a screw-retained crown. c) This follow-up bitewing radiograph shows stable bone levels around the implant five years after placement

Whether used for the replacement of a single tooth or multiple teeth, both implants and implant prostheses demonstrate excellent outcomes. Implant fixtures supporting single crowns have been shown to have ten-year survival rates of 95.2%, with implant-retained metal ceramic crowns exhibiting five-year survival rates of 98.3% (Fig. 11).^52^

## Considerations for dental implants

A range of considerations exist for dental implant treatment.^53^

These can broadly be divided into patient and clinical factors which can affect the initial osseointegration of the implant, long-term integration, or prosthesis survival.

### Patient factors

With respect to implant treatment, key factors which may impact initial healing include uncontrolled diabetes mellitus^53^ and uncontrolled periodontal disease.^54^

The risk of post-placement peri-implant bone loss are conditions such as a history of periodontal disease and current tobacco smoking.^55^

The use of ARDs for the management of osteoporosis or bony metastases have been shown to increase the risk of medication-related osteonecrosis of the jaws (MRONJ) and reduce implant survival in some circumstances.^51^ It is thought that low-dose ARDs (Table 5) pose little increased risk to early implant failure; however, the long-term effects are not fully understood.

**Table 5:** Typical therapeutic dosage of antiresorptive drugs. Reproduced with permission from B. Al-Nawas *et al*., ‘Group 3 ITI Consensus Report: Materials and antiresorptive drug-associated outcomes in implant dentistry', *Clinical Oral Implants Research*, 2023, vol 34, Wiley^51^

**Type of ARDs**	**Low-dose**	**High-dose**
Alendronate	70 mg/week per os	N/A
Risedronate	35 mg/week per os	N/A
Ibandronate	150 mg/week per os OR 3 mg/3 months i.v.	50 mg/day per os
Pamidronate	30 mg/3 months i.v.	90 mg/3-4 weeks i.v.
Zoledronate	5 mg/year i.v.	4 mg/3-4 weeks i.v.
Denosumab	60 mg/6 months s.c.	120 mg/3-4 weeks s.c.

Patients receiving high-dose ARDs, or those who have required surgical resection for MRONJ, are thought to be at higher risk of complications. In such patients, alternative treatment options should be considered alongside any other co-morbidities, and always with close cooperation with the ARD prescriber. Reference to guidelines such as those published by the Scottish Dental Clinical Effectiveness Programme also aid decision-making.

Some complications and risk factors are specific to implant treatment, such as osseointegration being compromised in smokers or those on high-dose ARDs. In such cases, the recommendation of a dental implant should be weighed carefully against retaining the tooth, if feasible, or an alternative prosthetic option.

### Clinical factors

#### Anatomical considerations

When planning implant placement, it is fundamental to consider the neighbouring anatomy to ensure that vital structures and adjacent teeth are not at risk of iatrogenic damage.

In the mandible, the mental foramen, inferior alveolar nerve, sublingual fossa and submental fossa all pose risks, such as permanent nerve damage or life-threatening bleeding. In the maxilla, consideration of the nasopalatine canal and maxillary sinus is imperative.

With the advent and increasing accessibility of CBCT, digital implant planning and guided surgery, implants can be planned and placed more precisely, thus reducing the risk of anatomical complications.^56^

In the posterior maxilla, one of the most frequent considerations is the requirement of sinus floor elevation, which increases treatment complexity and cost. The additional procedures may potentially be high-risk in some patients, such as those taking ARDs. When treatment planning in these patients, retention of the compromised root-filled tooth may be deemed preferable.

Similarly, implant placement in the mandibular second molar site may often risk perforation of the sublingual fossa and/or the inferior alveolar canal, meaning that tooth retention may again be favoured.

In the above clinical situations, an alternative may be to use short dental implants. These are defined as those less than or equal to 6 mm in length. Short dental implants have been shown to perform only slightly inferiorly than those over 6 mm (96% versus 98%).^57^ Accurately placing and achieving adequate primary stability can be challenging with shorter implants, but they may provide a viable alternative in cases where a tooth cannot be retained, and the anatomical risks are also high.

#### Aesthetic considerations

One of the most challenging aspects of implant treatment is achieving a good aesthetic outcome, and particularly, a natural soft tissue architecture.^58^ The goal is to achieve a harmonious and scalloped profile of the peri-implant mucosa, sufficient thickness of buccal mucosa and intact papillae.

When a tooth is lost, it is well-established that alveolar ridge resorption occurs,^59^ and this is more pronounced in sites where the buccal plate is thinnest,^60^ as a greater proportion of the bone is comprised of bundle bone (the alveolar bone that receives the periodontal ligament fibres, referred to as Sharpey's fibres). This resorption leads to a loss of contour of the soft tissues and although sufficient bone volume may remain to receive an implant, the aesthetic outcome can be compromised.

Thin and scalloped gingival phenotypes also pose further difficulties, as they are more susceptible to recession and/or translucency, thus risking discolouration from the underlying implant hardware.

In addition to accurate, prosthetically driven implant placement, various well-documented strategies have been proposed to overcome these aesthetic issues, including guided bone regeneration,^61^ soft tissue augmentation,^62^ immediate implant placement and socket shield, sometimes performed in combination.^63^ All of these protocols add additional complexity, cost and risk of complications to the course of treatment.

When replacing adjacent teeth, the soft tissue challenges are even greater, as more pronounced bone loss occurs and obtaining an optimal aesthetic result can be technically difficult.

The huge benefit of retaining a tooth is that it permits preservation of the osseous and associated soft tissue morphology. In highly challenging aesthetic cases, such as patients with a high smile line, a clinician may prefer to retain teeth to avoid adjacent implants or retain a root which can be submerged to create an aesthetically pleasing ridge contour for a pontic.^64^

#### Occlusal considerations

As implant occlusal failures are often mechanical in nature, exacerbated by the lack of a periodontal ligament, this makes the application of conventional occlusal principles challenging. Most of the data in relation to implant occlusion relies upon principles of engineering, clinical judgement and recommendations.^65^

**Fig. 12:**
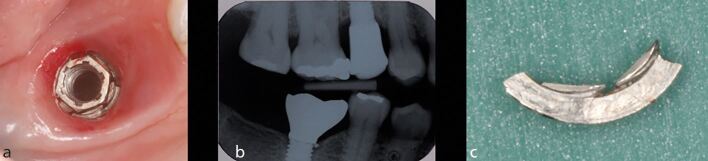
a) This patient presented with a loose implant crown in the right mandibular first molar site. The implant was placed 15 years previously, while the second molar tooth had been extracted two years ago. The patient had signs of attrition associated with parafunction. b) The associated radiograph showed a small radio-opaque fragment mesial to the implant head. Upon removal of the crown, multiple fractures of the implant shoulder were identified. A number of factors may have contributed to the fracture: 1) loss of the second molar increasing loading on the implant; 2) the slightly distal implant placement leading to non-axial loading of the implant; and 3) parafunction. c) The loose fragment that was removed from the implant

As with natural teeth, parafunction is a significant factor leading to complications of implant prosthesis (Fig. 12).^66^ Furthermore, it can lead to failure of integration in cases that are immediately loaded if the occlusion is not carefully managed.^67^ The presence of a parafunctional habit is an important predisposing factor for the failure of implant treatment as there is an increased risk of prosthesis fracture, with statistically significantly higher rates of mechanical and technical complications found in those exhibiting a parafunctional habit (60%) compared with those who do not (17%).^68^

For the above reasons, it is imperative to ensure that restorations are not exposed to excessive forces, and recommendations such as a mutually protected occlusion and light contacts in intercuspal position should be followed.

#### Biological complications

**Fig. 13:**
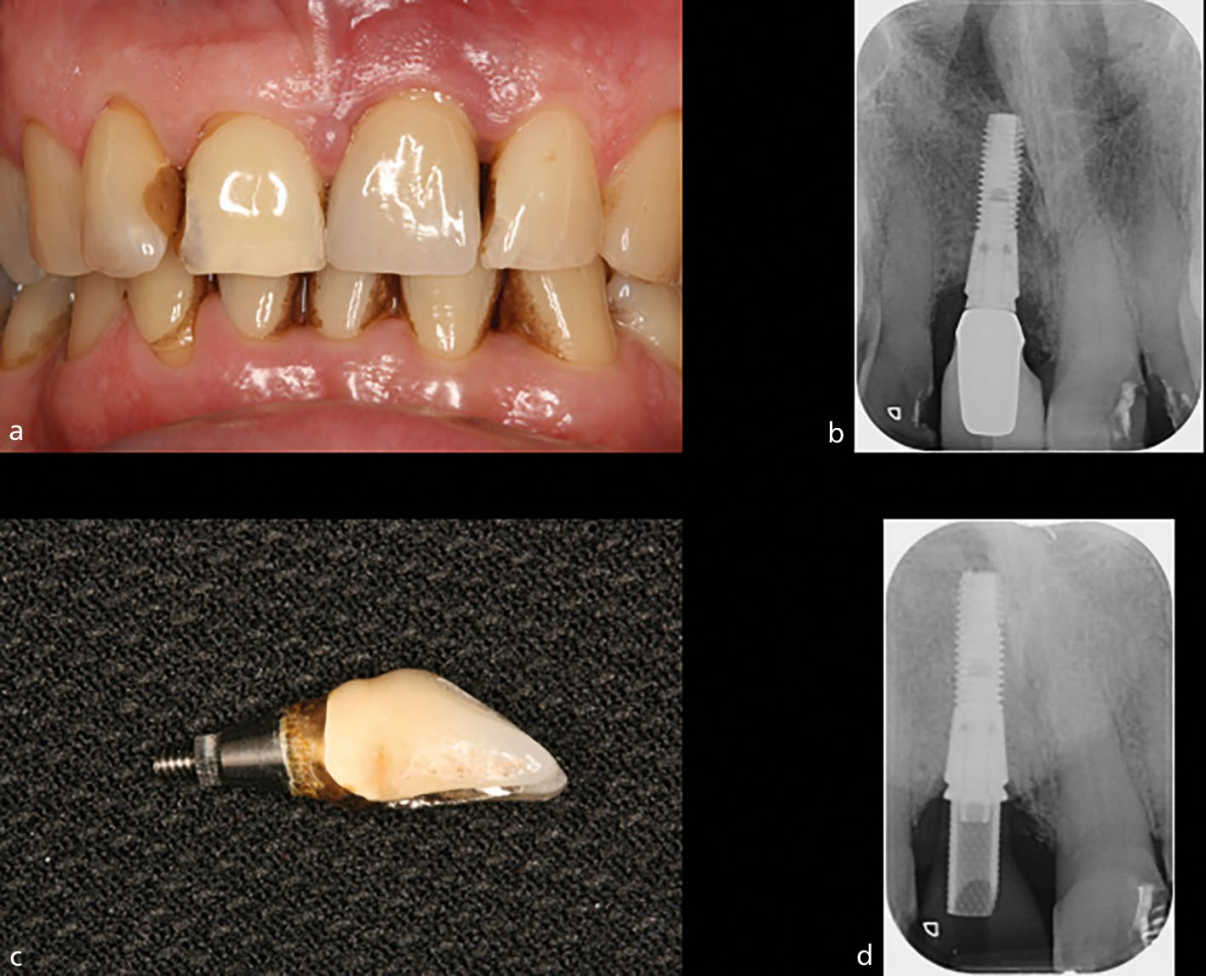
a) This patient presented with pain and suppuration from the labial mucosa of the maxillary right central incisor implant crown. The crown was cement retained and treatment had been provided several years previously. b) The radiograph showed bone loss around the implant head and an incompletely seated crown. c) Access was obtained to the screw and the crown was removed along with the abutment. Excess cement and incomplete seating of the crown was confirmed. d) A well-fitting provisional restoration was provided and bony healing was observed after two years

Peri-implant mucositis is an inflammatory condition of the soft tissues surrounding an implant in the absence of loss of supporting bone (Fig. 13).^68^ The clinical features are similar in many respects to that of gingivitis around teeth, and are characterised by inflammation, swelling and redness. The primary diagnostic features are bleeding on probing, which may be accompanied by increased probing depths, resulting from swelling, rather than bone loss. The main aetiological factor for peri-implant mucositis is plaque accumulation, with the host response to bacteria being modified due to conditions such as smoking and diabetes mellitus.

Peri-implantitis is characterised by inflammation of the peri-implant mucosa and progressive loss of the supporting bone. In addition to the features of peri-implant mucositis, peri-implantitis may present with mucosal recession and radiographic evidence of bone loss. Characteristically, peri-implantits will progress beyond the junctional or pocket epithelium. Patients at risk of developing peri-implantitis are those with poor plaque control, a history of periodontal disease, as well as a smoking habit. Submucosal cement, poorly fitting and/or contoured restorations may also predispose to both peri-mucositis and peri-implantitis.

Implants placed in augmented bone may have higher rates of peri-implantitis compared to those in pristine bone. The incidence of peri-mucositis is similar.^69^

## Comparative research

To assist both clinicians and patients in the decision-making process of the compromised root-filled tooth, studies comparing the treatment outcomes for natural teeth and implant replacement are useful. However, as discussed previously, it is imperative that the criteria used to evaluate both treatment modalities are similar, in order to make the conclusions meaningful. Furthermore, it is also essential to consider: a) that implant outcome studies, or for that matter, comparative studies, rarely, if ever, consider the need for preparatory surgery, such as grafting in the evaluation; and b) that there is a hugely different perspective when retaining a natural tooth as opposed to removing it and essentially ‘starting from scratch'. Patients may have an overriding desire to retain their own tooth rather than having an ‘artificial' replacement and will often consider heroic measures to maintain their natural dentition. For some patients, dental implant treatment may not be feasible for either medical, clinical or financial reasons. Therefore, when quoting success and/or survival rates to patients, these must be contextualised with respect to the specific case being treated, as they may be largely irrelevant for some patients.

**Fig. 14:**
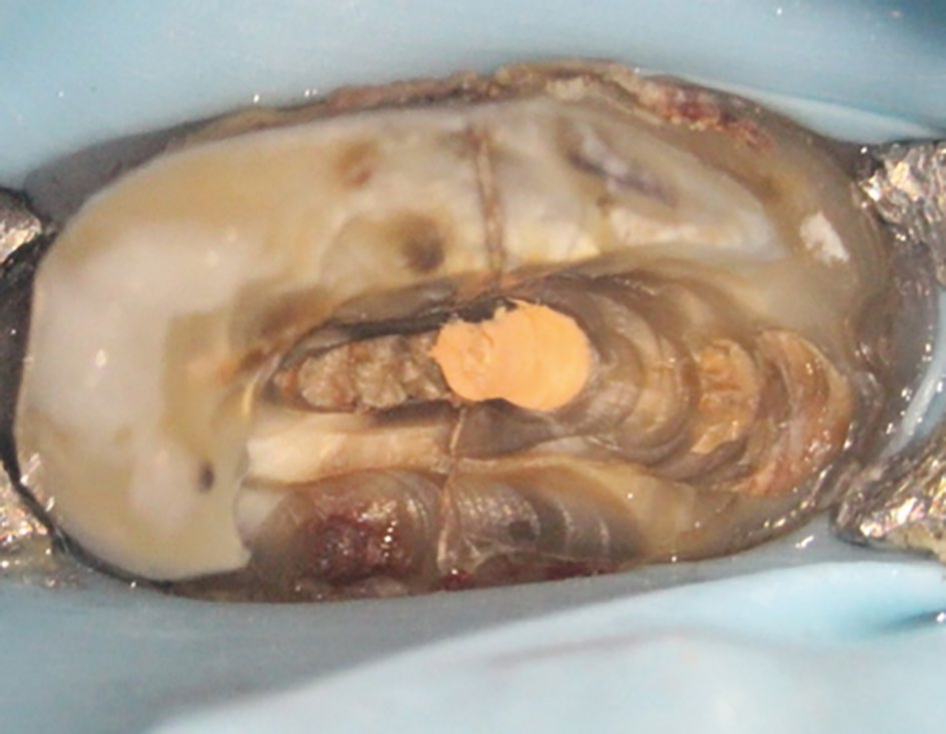
Recurrent caries and a deep crack have contributed to the failure of this compromised root-filled maxillary left first premolar tooth. Note how the recurrent caries has primarily affected the deeper restorative margins

Systematic reviews have compared both the success and survival of root-filled teeth and dental implants.^70^^,^^71^ The findings of these are variable and appear to be related to the chosen success/survival criteria and the observation period. Equivocal outcomes for natural teeth and implants have been reported by several authors.^70^^,^^72^ At 72 months there was no significant difference in survival rates, with both treatment modalities also having high success rates.^72^ However, a retrospective study of clinical records by Chatzopoulos *et al*. suggested superior outcomes for dental implants.^71^ Based purely on survival, and without analysis of non-terminal complications, root-filled teeth were shown to have a 72.7% survival rate after 77 months which was significantly lower (p <0.001) than that for implant treatment. Interestingly, in this analysis of over 13,000 patients, only increasing age and anxiety towards dental treatment were highlighted as risk factors, and this applied to both root-filled teeth and dental implants. It is important to acknowledge that the majority of implant failures occurred in the first year following placement (58.8%). However, the comparison of implant and root-filled tooth survival in this study is highly questionable. Firstly, the Multivariable Cox Regression Model does not record any clinically relevant factors, such as oral hygiene, periodontal disease, caries activity, bruxism, or carious/non-carious tooth structure failure (Fig. 14). Secondly, implants were placed by residents or faculty members, presumably in carefully selected cases, whereas endodontic treatment was undertaken by dental undergraduate students, presumably in much less carefully selected cases, as indicated by the significantly higher number of root-filled teeth included in the study (8,915 versus 4,519 implants). A matched comparison of root-filled teeth and dental implants which included relevant clinical factors would likely have yielded different results. Case selection bias is a major problem in most studies comparing the survival and success of root filled teeth and dental implants. For example, single-unit dental implants are rarely placed to replace second molars, whereas all survival studies on root-filled teeth include large numbers of second molars, which, as discussed previously, are well-known to be associated with the lowest survival rate compared to other tooth types.

Highlighting the greater prevalence of early implant complications, a cross sectional study comparing the outcomes for 196 single-tooth implants with the same number of root-filled teeth were, respectively, as follows: success = 73.5% and 82.1%; survival with no intervention = 2.6% and 8.2%; survival with intervention = 17.9% and 3.6%; and failure = 6.1% and 6.1%.^73^ A further analysis of the same cohorts of patients identified smoking as a risk factor for both groups. Albeit far less prevalent than for single-tooth implants, biological complications were the primary cause of early failures in root-filled teeth.^74^

With specific regards to root canal retreatment when compared to dental implant treatment, there is a lack of research. However, one small randomised controlled trial with a three-year follow-up found no difference in the survival of teeth having undergone root canal retreatment and single-tooth implants.^75^ It was also found that it took significantly more days (191 days), to complete the implant rehabilitation compared with the treatment of a root-filled tooth (61 days). However, the chair time was much greater for endodontic-restorative procedures than for implant treatment (629 versus 326 minutes). At the three-year follow-up, the soft tissue aesthetics were superior for root-filled teeth than they were for dental implants.

Systematic reviews have also attempted to compare the outcomes for root canal treatment, retreatment, root-end surgery and dental implants.^76^^,^^77^ Both reviews identified favourable outcomes for root canal treatment, retreatment and dental implants. However, root-end surgery was found to have inferior outcomes in both analyses. Elemam and Pretty published survival rates of 86.02% for root canal treatment, 78.2% for root canal retreatment, 63.4% for root-end surgery and 90.9% for dental implant treatment with an observation period of 6.7-8.7 years.^76^ However, it should be acknowledged that one of the included studies in relation to root-end surgery had a success rate of only 27.84% as the treatment was not carried out using contemporary microsurgical techniques; this significantly lowered the survival rate of this treatment modality and the results are not consistent with the high success rates observed in numerous studies where microsurgical techniques have been used. In a further review, there were no considerable differences in survival rates for all four treatment modalities when observing outcomes up to ten years.^78^ However, it was noted that single tooth dental implants had slightly superior outcomes which became more evident after ten years of follow-up.

In a recent systematic review comparing the survival of root-filled teeth (after both initial and secondary treatment) with that of dental implants, three studies reported comparable survival rates for both modalities over the first three years.^79^ However, after three years, the survival rate of root-filled teeth decreased, with a higher failure rate compared to implant-supported restorations. The findings of the review were associated with the inclusion criteria from two studies which included teeth which had undergone all types of endodontic procedures (primary treatment, retreatment and root-end surgery). In addition, teeth with and without definitive cuspal coverage restorations were also included. Root-filled teeth that did not receive cuspal coverage had earlier failures compared with those that did. Interestingly, three included studies reported lower survival rates for implant-supported prostheses.

## Conclusion

Overall, there is a lack of long-term prospective data comparing the success and survival of root-filled teeth and dental implants. With careful planning and well executed treatment, both options offer favourable long-term outcomes. Decision-making for the compromised tooth should be based on consideration of both non-clinical and clinical factors. Often, there is no ‘ideal' treatment option and both root-filled teeth and dental implants can fail both biologically and biomechanically. Occlusal and non-occlusal forces are an important factor contributing to the failure of both treatment modalities and should be carefully assessed during both planning and management.

## Data Availability

This study used third party data which has either been made available under licence that the author does not have permission to share, or is available from the relevant corresponding authors (as per the citations) upon reasonable request. Data sharing is not applicable to this article as no new data were created or analysed in this study.
