# Correction to: Endodontic and dental implant treatment: key considerations and comparisons

**DOI:** 10.1038/s41415-025-8864-3

**Published:** 2025-06-13

**Authors:** Bhavin Bhuva, Pareet Shah, Francesco Mannocci

**Affiliations:** 989932331190704492720 Honorary Clinical Lecturer, King´s College London, UK; Consultant in Endodontics, Guy´s Hospital, Guy´s and St Thomas´ NHS Trust, UK; Specialist in Endodontics, Private Practice, United Kingdom; 032136714895314408729 Senior Clinical Lecturer and Deputy Director Implant MSc, UCL Eastman, London, UK; Specialist in Prosthodontics, Private Practice, United Kingdom; 900627091276899225506 Professor and Head of Endodontology, King´s College London, UK; Specialist in Endodontics and Restorative Dentistry, Private Practice, United Kingdom

Journal's correction note:

Review *Br Dent J* 2025; **238:** 779–791.

When this Review was originally published, the super title was incorrect. The super title was published as ‘Literature Review' but should have been ‘Expert Review'.

The journal apologises for any inconvenience caused.

